# Sustainable Waterborne Polylactide Coatings Enabled by Hydrophobic Deep Eutectic Solvents Plasticization

**DOI:** 10.3390/polym18020154

**Published:** 2026-01-07

**Authors:** Denys Baklan, Victoria Vorobyova, Olena Sevastyanova, Taras Karavayev, Oleksiy Myronyuk

**Affiliations:** 1 Department of Chemical Technology of Composite Materials, Chemical Technology Faculty, Igor Sikorsky Kyiv Polytechnic Institute, Beresteiskyi Ave. 37, 03056 Kyiv, Ukraine; o.myronyuk@kpi.ua; 2 Physical Chemistry Department, Chemical Technology Faculty, Igor Sikorsky Kyiv Polytechnic Institute, Beresteiskyi Ave. 37, 03056 Kyiv, Ukraine; vorobyovavika1988@gmail.com; 3 Division of Wood Chemistry and Pulp Technology, Department of Fiber and Polymer Technology, KTH Royal Institute of Technology, Teknikringen 56-58, 100 44 Stockholm, Sweden; 4 Commodity Science and Customs Affairs Department, State University of Trade and Economics, Kyoto St., 19, 02156 Kyiv, Ukraine; t.karavayev@knute.edu.ua

**Keywords:** polylactide, polymer, water dispersion coatings, polymer film, coating, glass transition temperature

## Abstract

This work presents an approach to water-dispersible polylactide (PLA) particle fabrication and their application in low-temperature film formation using a combination of mechanical dispersion and ultrasonication techniques. Stable PLA dispersions were obtained after removal of surfactant and allowed for thin-film preparation, exhibiting a significantly reduced minimum film formation temperature (MFFT) from 128 °C to 80 °C after reducing the characteristic particle size from ~2.2 µm to ~140 nm. To tailor the interfacial behavior and mechanical flexibility of the resulting coatings, a set of conventional and bio-based plasticizers was evaluated, including epoxidized fatty acids, PEG-400, and several hydrophobic deep eutectic solvents (HDESs) synthesized from menthol and carboxylic acids. Compatibility between PLA and each plasticizer was predicted using Hansen solubility parameters. The efficiency of plasticization was assessed through glass transition temperature suppression in solvent-cast films. The combination of submicron PLA particles and selected plasticizers enabled film formation at temperatures as low as 48 °C, confirming the potential of these systems for energy-efficient coating technologies. Furthermore, composite coatings incorporating micro-sized cellulose fibers (L/D ≈ 10.5–11.5) regenerated from agricultural residues were successfully obtained, demonstrating the feasibility of integrating bio-derived fillers into waterborne PLA formulations. In this study, the use of water-insoluble deep eutectic solvents type plasticizers for PLA coatings from water dispersions was reported for the first time. This establishes a foundation for developing sustainable, low-VOC, and low film formation temperature PLA-based coating materials.

## 1. Introduction

The transition from fossil hydrocarbon-based polymers to bio-based and optionally biodegradable materials is one of the significant routes for reducing carbon footprint of plastic, composites, packaging (including high-volume food and personal care), and coatings industries [1,2]. Contemporary, industrially established bioplastics include polylactide (PLA), microbial polyhydroxyalkanoates (PHAs; e.g., PHB/PHBV), and poly(butylene succinate) (PBS), with polyethylene furanoate (PEF) emerging for packaging applications. Authoritative overviews detail their production routes, properties, and markets [3,4,5,6,7,8]. Many leading bioplastics, especially PHAs, are synthesized intracellularly by bacteria via fermentation and recovered from biomass rather than by synthetic emulsion polymerization, which explains why “emulsion routes” are generally inapplicable to their primary manufacture [9]. We focus our research on PLA because it couples a mature, large-scale supply chain, robust melt processability and property tunability, and broad end-of-life options at competitive cost [3,10]. One possible approach to producing coatings from such bioplastics is the use of their solutions in organic solvents. However, this strategy conflicts with the principles of green chemistry, specifically the reduction in volatile organic compound emissions, as well as with relevant regulatory frameworks (i.e., Industrial Emissions Directive 2010/75/EU). Using aqueous dispersions is the most promising strategy for addressing this challenge. Such dispersions can be obtained from polymer solutions, for example via the solvent evaporation method. This approach is particularly advantageous for polymers such as PLA or PBS because it allows dispersion formation under mild conditions without subjecting the polymer to thermal degradation, while also enabling high solids content and narrow particle size distributions. Concentrated PLA and PHA dispersions (20–40 wt. %) have been successfully prepared through controlled solvent evaporation, demonstrating good film-forming ability and stability [11,12,13]. Importantly, all handling of organic solvents occurs at the production stage rather than during coating application, ensuring effective capture, recycling, and regulatory compliance under industrial emission control standards. 

Most bio-based polymers have relatively high glass transition temperatures, which makes them brittle materials unsuitable for direct film formation [14]. To overcome this disadvantage, plasticizers are used, which, by replacing intermolecular interactions of the polymer with interactions with more mobile molecules, increase the segmental mobility of polymers and, consequently, reduce the brittleness of the material [15,16]. The most common plasticizers used in PLA formulations intended for extrusion are polyethylene glycol (PEG) [17], citrate esters, especially acetyl tributyl citrate (ATBC) and triethyl citrate (TEC) [18], oligomeric lactic acid, etc. [16]. Ionic liquids are promising plasticizers for polylactide [19] because of their highly tunable polarity, low volatility, and strong specific interactions with ester groups [20,21,22]. In addition to compatibility with the polymer, plasticizers used in water dispersion coating technology must also be insoluble in the aqueous phase [23,24,25]. On the one hand, this will ensure uniform film formation, and on the other hand, it prevents plasticizer migration when in contact with moist environments. Therefore, plasticizers such as PEG are unsuitable for these applications.

An additional feature of polymer dispersion coating technology is the process of film formation through the fusion of dispersion particles. As shown by Arjmandi et al. [26], particle fusion occurs when the dispersion medium (water) evaporates due to the capillary pressure that arises between the particles. The capillary pressure magnitude depends on the capillary diameter. However, capillary pressure is not all that is needed to form a film, the polymer particle material itself must be soft enough to deform due to the mobility of macromolecular segments. This softness depends on the ambient temperature and determines the minimum temperature at which the film is formed. The value of this temperature is always higher than the glass transition temperature of the material. Obviously, to use polylactide dispersions as a substitute for traditional water-dispersible binders, this temperature should be below 5 °C. An alternative may be an intermediate technology that partially uses additional heating for coalescence, which is widely used in powder paint [27]. 

An additional factor determining the applicability of dispersions is the possibility of compounding them with various functional additives, such as reinforcing fillers and pigments, which must form a monolithic structure with the film-forming phase after curing.

The problem of this study is the need to obtain such aqueous polylactide dispersions that can form a film at temperatures as close as possible to the ambient temperature and can be compounded. To solve this problem, the influence of plasticizers, including promising deep eutectic solvents, on the reduction in the glass transition temperature and the minimum film formation temperature, as well as the reduction in these indicators by decreasing the particle size, is investigated. The work also demonstrates the formation of composites based on film formers and reinforcing cellulose fillers from agricultural waste.

In this work, it was demonstrated for the first time that hydrophobic deep eutectic solvents can function as effective plasticizers for PLA-based coatings formed from aqueous PLA dispersions, thereby extending plasticization concepts toward waterborne, technologically, and environmentally preferable systems. The novelty also lies in introducing the plasticizer directly at the dispersion stage, enabling control over compatibility and film formation during drying. In addition, we systematically examine the formation of continuous PLA coatings in the presence of cellulose-based fillers, outlining pathways for further formulation optimization and practical use. 

## 2. Materials and Methods

**Figure 1:**
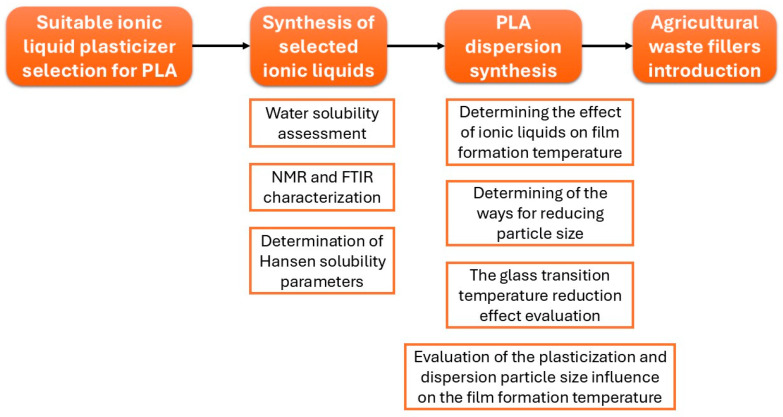
Research structure scheme.

The research structure (Figure 1) included an analytical selection of the most suitable polylactide plasticizers from the class of deep eutectic solvents, followed by the synthesis of selected samples, for which water solubility was experimentally evaluated. For the synthesized samples and benchmark plasticizers, the effect on glass transition temperature reduction was determined. The next important stage was to determine the possibility of reducing particle size by modifying the production modes (mechanical dispersion and ultrasonication). Next, the influence of two main factors (plasticization + dispersion particle size) on the film formation temperature was considered. The final stage of the work was to determine the possibility of introducing reinforcing fillers into the polylactide films formed from the dispersion. 

### 2.1. Materials

The Ingeo Biopolymer 4060D (NatureWorks, Minnetonka, MN, USA) polylactide (PLA) was used in this work. This PLA grade was selected because its amorphous structure produces a relatively low glass transition temperature (55–60 °C), which improves its suitability for use in films. A few deep eutectic solvents were selected as plasticizers: menthol–levulinic acid 1:1 (MenLev), menthol–acetic acid 1:1 (MenAc), menthol–lactic acid 1:1 (MenLac), menthol–oleic acid 1:1 (MenOl), and menthol–linalool 1:1 (MenLil). Epoxy oleic acid, epoxy linoleic acid, and PEG-400 were chosen as references. Epoxy oleic acid and epoxy linoleic acid were synthesized according to the methodology described in our previous paper [28]. Dichloromethane was used as a solvent for PLA (Thermo Fisher Scientific, Waltham, MA, USA).

### 2.2. Obtaining Polylactide Dispersions

**Figure 2:**
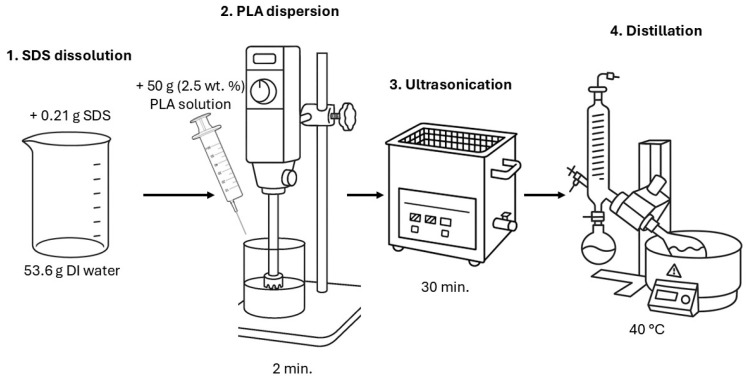
Polylactide dispersion preparation procedure.

The method for obtaining the PLA dispersion consisted of the following steps (Figure 2). First, 0.21 g of sodium dodecyl sulfate (SDS) was dissolved in 53.6 g of distilled water using a top stirrer. Next, after complete dissolution, 50 g of a 2.5 wt. % solution of PLA 4060D in dichloromethane was gradually added to the solution. Mixing at this stage was performed using a WiseTis HG 15A high-speed disperser (Daihan Scientific, Daejeon, Korea) at a speed of 27,000 rpm for 2 min. The dispersion was then transferred to an ultrasonic bath and subjected to ultrasonic treatment for 30 min, with the dispersion temperature maintained below 25 °C to minimize dichloromethane evaporation. The treatment parameters were 35 kHz and 50 W. 

Efficient removal of dichloromethane was performed using an RV 3 V rotary evaporator (IKA, Staufen, Germany). The water bath temperature was set to 40 °C. A slight vacuum was applied to accelerate the removal of dichloromethane. The rotary evaporator vacuum treatment was stopped when water began to be removed. After this step, the dispersions were cooled to room temperature and vacuumed under static conditions for an additional 2 h. The combination of high volatility, reduced pressure, extended vacuum drying, and thin-film drying ensures that any residual dichloromethane in the final PLA particles and coatings is expected to be negligible. Dichloromethane was used because of its high evaporation rate. The resulting particles were separated by centrifugation (13,500 rpm for 20 min, CF-10 Daihan Scientific, Wonju, Korea) 3–4 times. The residual surfactant was assessed by measuring the surface tension of the centrifuged liquid (pendant drop technique on BGD-190, Biuged Precision Instruments, Guangzhou, China); centrifugation was finished when the value was higher than 72.0 ± 1.2 mJ/m^2^. The obtained particles were further dried on a glass substrate at 20 °C and RH less than 20%. This resulted in nanometer-sized PLA particles suitable for further modification and use in film materials. A vacuum drying oven (SV-80, UOSlab, Kyiv, Ukraine) at a temperature of 40 °C and full vacuum was used to concentrate the PLA dispersion for rapid water removal.

### 2.3. Obtaining Biofillers

Agricultural waste was used to obtain biofillers. Initially, cornstalks and sunflower shell hulls were cut into flakes up to 1 mm long, and coconut shells were first crushed in a jaw crusher and then, when the particle size reached several millimeters, further crushed in a hammer crusher. The chemical treatment of the fibers was the same for all materials. The fillers were treated with a mixture of acetic acid and hydrogen peroxide in a volume ratio of 70:30 for two hours at a temperature of 95 °C, after which they were cooled. They were then immersed in a 0.1 M potassium hydroxide solution for two days at room temperature, after which they were treated with hydrogen peroxide. After chemical treatment, the materials were passed through an FDM-Z-150 colloid mill (Vektor, Guangzhou, China) and then through a blender. The ground particles were sieved through sieves under running water between sieves with 63 µm (lower) and 400 µm (upper) openings. Polymethylhydrosiloxane (XIAMETER MHX-1107, Dow Chemical, Midland, MI, USA) was used to hydrophobize the fillers. For this purpose, the fibers were placed in a 1 wt. % solution of polymethylhydrosiloxane for 3 h. The fibers were then washed with xylene and kept at a temperature of 130 °C to fix the siloxane on the surface of the cellulose fibers.

### 2.4. Characterization Methods

For particle characterization, optical microscopy was used (Konus Academy optical microscope with UCMOS 1300 digital camera (Sigeta Optics, Kiev, Ukraine; ver. 4.11.23945.2023.1121, ToupTek, Hangzhou, Zhejiang, China). The surface topography of the obtained films was examined using an MIRA3 SEM (Tescan, Brno, Czech Republic).

Differential scanning calorimetry (DSC) was performed using a PT1000 DSC (Linseis, Selb, Germany). Samples (~5–10 mg) were placed in aluminum pans and measured under nitrogen purge of 30 mL/min. The thermal program consisted of first heating from 20 to 200 °C at 10 °C/min, cooling to 20 °C at 10 °C/min, and second heating to 200 °C at 10 °C/min. Glass transition temperature (T_g_) was determined from the second heating curve as the midpoint of the heat-capacity step after baseline correction performed using the automatic baseline function of the analysis software—Origin Pro ver. 2024 (OriginLab Corporation, Northampton, MA, USA). 

The minimum film formation temperature (MFFT) of PLA dispersions was determined using a temperature-controlled heating plate coupled with optical microscopy (reflected light). Dispersions were applied onto glass microscope slides using a film applicator with a 500 µm gap (wet film thickness). The coatings were dried at room temperature and RH ≈ 50% to obtain films with a dry thickness of 60–100 µm. Then, the samples were then heated from room temperature in 1 °C steps with a 0.5 min dwell per step. The “transparency point” was used as the operational criterion for film formation: MFFT was defined as the lowest substrate temperature at which the coating became optically continuous and transparent under the microscope, with disappearance of visible particle boundaries. For each formulation, measurements were performed three times on independently prepared films.

For deep eutectic solvent analysis, ^1^H NMR was used. HDESs were dissolved in a deuterated solvent to prepare the NMR samples. Chemical analysis was performed via ^1^H NMR spectroscopy (400 MHz, Bruker Avance III HD 400 MHz, with a 5 mm broadband probe). Each spectrum was recorded with 120 scans and 48 K data points. To confirm the theoretically predicted interactions through functional reactive groups of the compounds, FTIR spectra were obtained using a IRSpirit spectrometer (Shimadzu, Kyoto, Japan) with a spectral resolution of 4 cm^−1^ in the range of 400–4000 cm^−1^. FTIR spectroscopy in ATR mode (IRSpirit, Shimadzu, Kyoto, Japan) was used for PLA film analysis.

Hansen solubility parameters were used to model the compatibility of plasticizers with polymers. Hansen’s theory includes three interactions between molecules: δ_d_ (dispersion forces energy), δ_p_ (dipolar intermolecular forces energy), and δ_h_ (hydrogen bonding energy). The HSP is measured using the sphere method, where the center of the sphere has the HSP coordinates of the polymer under study, and the radius determines the limits of compatibility. According to the Hansen model, the radius ratio between the plasticizer (R_a_) and polymer (R_o_) spheres is a condition for their compatibility. The Relative Energy Difference (RED) parameter is the ratio of R_a_/R_o_ (if RED > 1, the plasticizer is incompatible with the polymer; if RED < 1, it is compatible).

Hansen solubility parameters for deep eutectic solvents were determined using a set of solvents (water, dichloromethane, isopropyl alcohol, hexane, ethyl acetate, n-butanol, cyclohexane, dimethylformamide, o-xylene, and dimethyl sulfoxide) or their mixtures. For the experiments, dichloromethane was used as the solvent. All named substances were purchased from the local supplier HLR Ukraine (Chemlaborreactiv LLC, Brovary, Ukraine).

The solubility borders and sphere centers in D, P, and H coordinates (Hansen sphere calculation) were calculated using the HSP Excel spreadsheet prepared by Dr. Diaz de los Rios [29,30].

## 3. Results and Discussion

The Hansen solubility parameters were employed to predict the compatibility of the deep eutectic solvents with the biopolymers. The source of data for deep eutectic solvents was the publication by Fernandes et al. [31], which contains these parameters defined using empirical and semi-empirical approaches. As the authors conclude, for the dispersion component δ_d_ all approaches give close values; for δ_p_, the Hoftyzer and Van Krevelen model provides the best accuracy; and δ_h_ can be predicted directly only with a large error. Thus, it is recommended to calculate the last parameter using δ_t_, which can be performed using Equation (1):

(1) $$\delta_{h}=\sqrt{\delta_{t}^{2}-\delta_{d}^{2}-\delta_{p}^{2}},$$

From the full set of HDES, menthol–levulinic acid (1:1) and menthol–acetic acid (1:1) were selected as nonpolar candidates. Although solubility parameters for menthol–lactic acid (1:1) were not found in the literature, its presumed low polarity and the affinity of the hydrogen bond acceptor (lactic acid) for the monomeric unit of the polymer to be plasticized justified the inclusion of this HDES in the analysis. All synthesized HDES were tested for water solubility, and it was surprisingly found that, despite the relatively nonpolar hydrogen bond donor menthol, they are water-soluble. As mentioned previously, this property may cause leaching of the plasticizer in moist environments (especially from thin-layer films); moreover, the plasticizer solution in water may be absorbed by porous substrates after application, resulting in a decrease in the PLA/plasticizer ratio. To overcome this issue, two water-insoluble plasticizers from our previous work were utilized (epoxidized oleic and linoleic acids), and an additional HDES, menthol–oleic acid (1:1), was synthesized. This HDES is not water-soluble and forms dispersions under mechanical stirring, which may be stabilized with SDS (like PLA dispersions). PEG-400 was included in the analysis as a benchmark plasticizer.

### NMR and FTIR Characterization of HDES

For hydrophobic deep eutectic solvents (HDESs), ^1^H NMR spectroscopy was selected due to its ability to track the presence and strength of hydrogen bonds, the degree of association between the hydrogen bond donor and acceptor, and possible conformational changes. For HDES, which often include nonpolar and weakly interacting molecules, ^1^H NMR can detect subtle shifts in the proton environment that indicate the formation of a eutectic network. This information is essential for understanding the stability, miscibility, and physicochemical behavior of HDES. FTIR spectroscopy complements ^1^H NMR because it provides direct evidence of interactions between specific functional groups that NMR cannot fully reveal. While ^1^H NMR allows changes in the electronic environment of protons to be determined, FTIR detects vibrations associated with hydrogen bonds in –OH, –NH, and C–H groups. 

**Figure 3:**
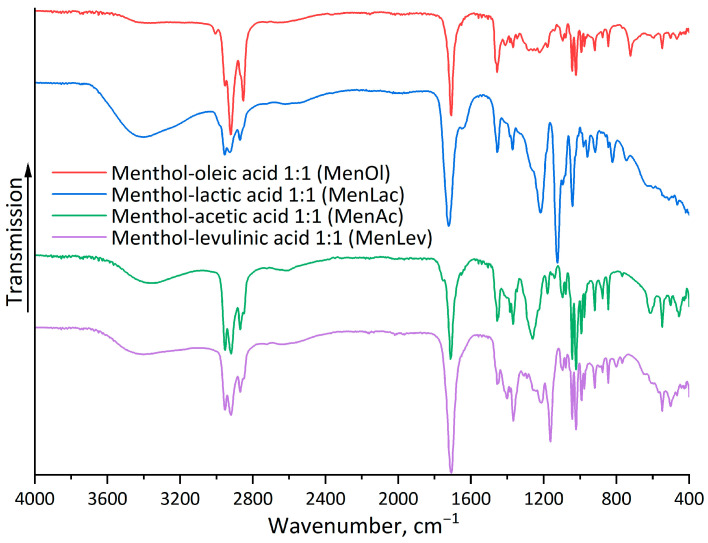
FTIR spectra of HDES/menthol–oleic acid 1:1 (MenOl); menthol–lactic acid 1:1 (MenLac); menthol–acetic acid 1:1 (MenAc); menthol–levulinic acid 1:1 (MenLev).

Analysis of the FTIR spectra (Figure 3) of HDES based on menthol–levulinic acid, menthol–acetic acid, menthol–lactic acid, and menthol–oleic acid shows a broad O–H stretching band around 3600–3200 cm^−1^, indicating the presence of a hydrogen bond between the hydroxyl group of menthol and the carboxyl group of organic acids. The high-intensity peak at 1710–1730 cm^−1^ indicates stretching vibrations of the C=O bonds of the carboxyl group in the acid. Additionally, the FTIR spectrum shows peaks corresponding to C–O bonds at 1180–1040 cm^−1^ and C–H bonds at 2910–2930 cm^−1^.

**Figure 4:**
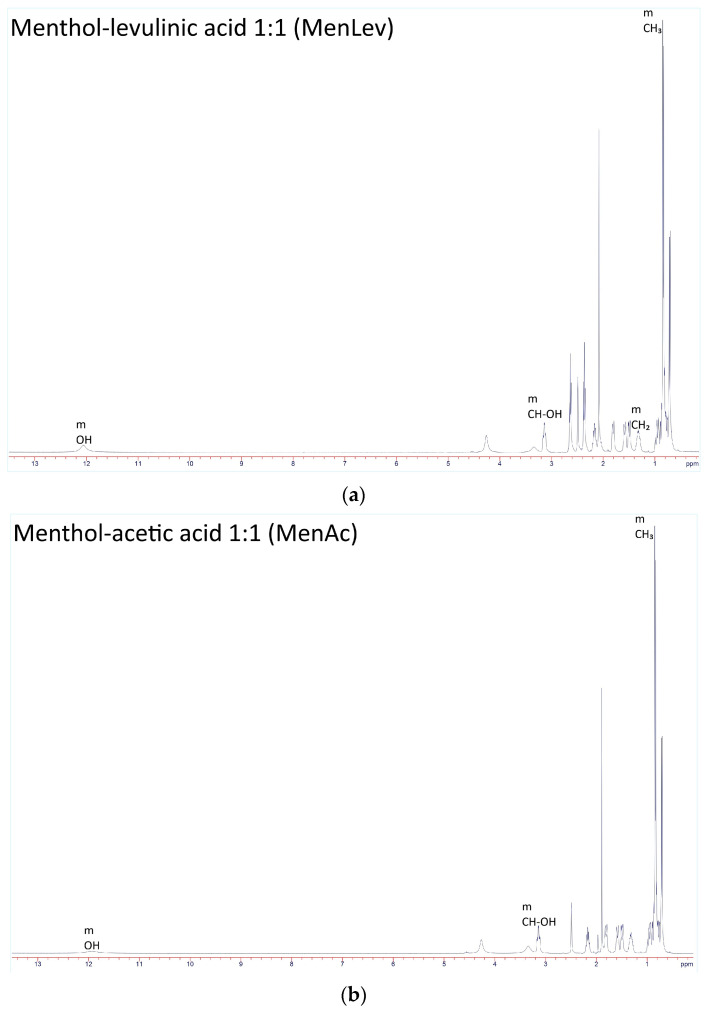

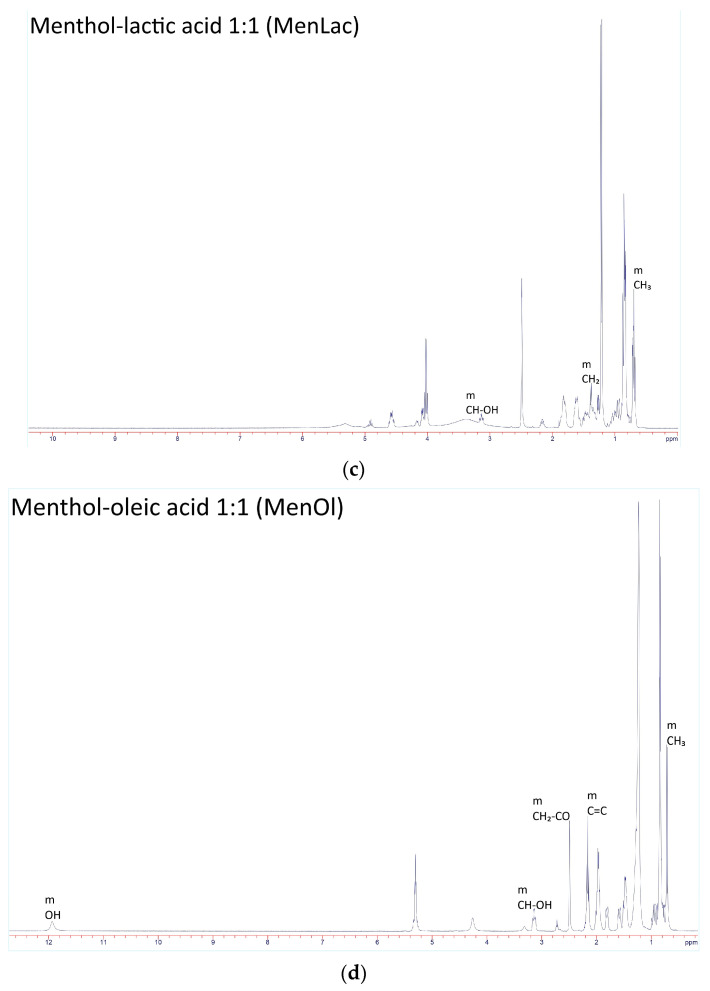
^1^H NMR spectra of HDES: (**a**) MenLev; (**b**) MenAc; (**c**) MenLac; (**d**) MenOl.

For all obtained HDES, ^1^H NMR spectra (400 MHz, CDCl_3_) (Figure 4) showed a series of signals at δ 0.80–1.10 (m, CH_3_ menthol), 1.20–2.20 (m, CH_2_). For menthol–levulinic acid, peaks at 2.45 (t, CH_2_–CO), 3.35 (m, CH–OH), 4.10 (m, CH–O), and 9.70 (s, COOH) are present, which is consistent with the proposed composition and confirms the formation of hydrogen bonds in the HDES. Similarly, for HDES menthol–acetic acid, there are peaks at 2.08 (s, CH_3_CO), 3.35 (m, CH–OH), 4.10 (m, CH–O), and 11.30 (s, COOH), while for HDES based on menthol–lactic acid, peaks at 3.35 (m, CH–OH menthol), 4.15 (q, CH–OH lactic acid), and 9.75 (s, COOH) are detected. Finally, for the DES based on menthol and oleic acid, signals are observed at δ 0.88 (t, 3H, CH_3_ oleic acid), 1.20–1.35 (m, (CH_2_)_n_ oleic chain), 1.98–2.05 (m, allyl CH_2_ adjacent to C=C), 2.30–2.40 (t, ^2^H, CH_2_–CO oleic acid), 3.30–3.40 (m, CH–OH menthol), 4.05–4.15 (m, oxygenated menthol methine, if present), 5.30–5.40 (m, ^2^H, vinyl CH of oleic acid), and 10.8–11.8 (broad s, COOH, hydrogen bond).

To assess the compatibility between all selected and synthesized plasticizers and polylactide, HSP calculations were performed (Table 1). As can be seen, the RED values of all pairs (plasticizer—PLA) are less than 1, indicating compatibility. It may also be noted that the lowest values of RED are shown for epoxidized oleic and linoleic acids, while the highest values are found for MenAc and MenLac, respectively. On the other hand, the benchmark PEG-400 has a moderate RED value. 

**Figure 5:**
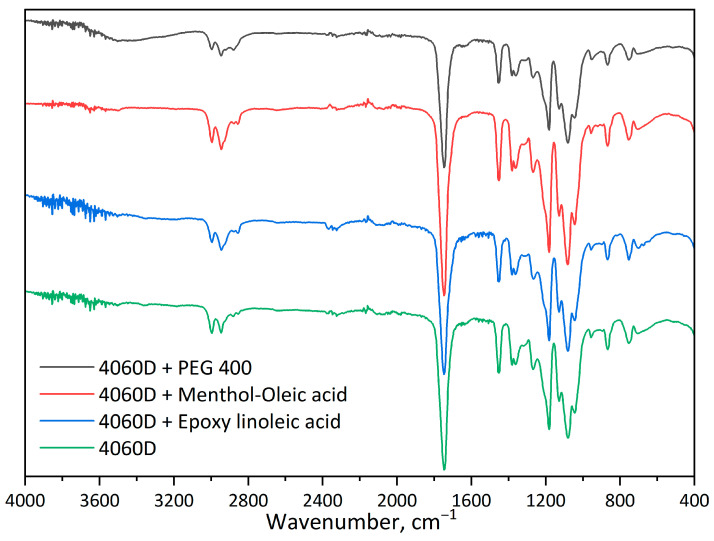
FTIR spectra of PLA–plasticizer compositions.

FTIR spectroscopy revealed no significant changes in the chemical composition (Figure 5), confirming the absence of covalent interactions between the polymer and the plasticizer.

**Figure 6:**
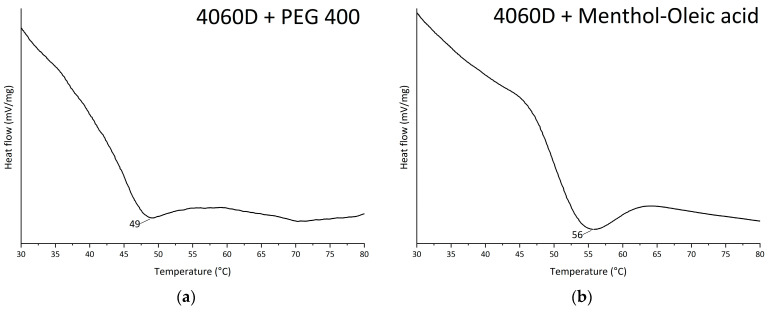

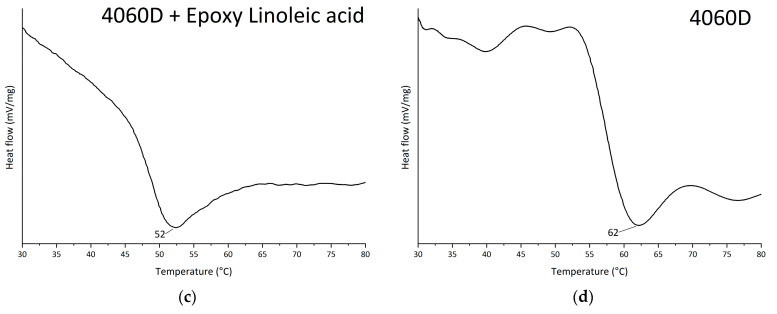
DSC results for PLA and its plasticizers: (**a**) 4060D plasticized with 20 wt. % PEG-400; (**b**) 4060D plasticized with 20 wt. % MenOl; (**c**) 4060D plasticized with 20 wt. % epoxy linoleic acid; (**d**) neat 4060D.

The effectiveness of a plasticizer is commonly assessed by the grade of depression of the polymer glass transition temperature. The nature of this effect lies in the increased segmental mobility of polymer molecules when a compatible, lower-molecular-weight plasticizer is introduced between the chains or their supramolecular arrangements [33]. In this work, T_g_ suppression was determined using DSC (Figure 6) on polymer–plasticizer (20 wt. %) mixtures, which were cast as films from dichloromethane solutions and, after complete drying at 50 °C for 1.5 h, and cooled to room temperature in a dry chamber. The T_g_ of the 4060D polymer was 62 °C, which is slightly higher than the interval stated in the TDS (55–60 °C) but is consistent with the value measured in [34]. 

PEG-400 effect is the most significant among all the plasticizers considered: the T_g_ lowers by 17 °C; in case of epoxidized linoleic acid, the decrease is 10 °C, and the MenOl gives only 6 °C. Such T_g_ suppression only generally correlates with the HSP affinity prediction; the substances considered are effective in T_g_ suppression, indicating the approximation of such analysis. A similar conclusion was found by Xuan et al. [35], where it was shown that HSP may be an indicator of miscibility in general; however, structural factors play a crucial role in T_g_ suppression. Mascia et al. [36] described the phenomenon of antiplastification as considered for the highly compatible pairs of polymer–plasticizers, again accounting on the structure features of interacting molecules. Ruiz et al. [37] showed that the affinity predicted by HSP is true for a certain plasticizer content range.

**Figure 7:**
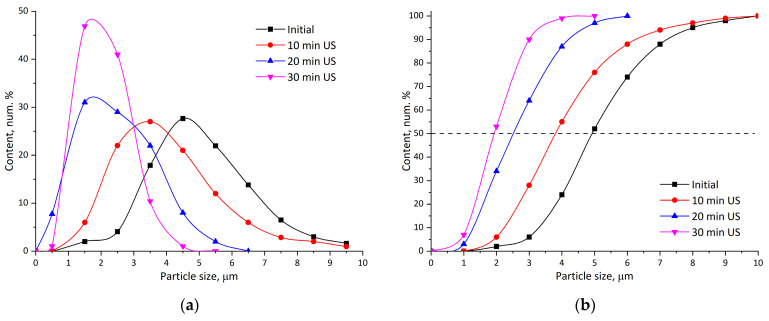
Distribution of emulsion particle size: (**a**) differential; (**b**) cumulative.

The PLA particle formation by the solvent evaporation method was performed using the mechanical dispersion of the solution of PLA in dichloromethane in water, supported with the addition of SDS surfactant and additional ultrasonication. The effectiveness of ultrasonication is determined by the dispersed phase viscosity balance and the energy density, which is provided by the ultrasonic homogenizer. In our experiments with a low-power homogenizer (60 Wt), it was noted that only at a polymer concentration of 2.5 wt. % was the submicron fraction formed (Figure 7). As stated by Ruiz et al. [37], at higher ultrasonic energy density, it is possible to homogenize more concentrated solutions. As can be seen from Figure 7, mechanical stirring alone is able to deliver particles with an average diameter of 4.5 μm, while ultrasonication decreases this value to 1.5 μm. Together with this, the submicron fraction appears. 

As was recently shown by the Belletti group (Buoso et al.) [38], using a combination of mechanical dispersion + ultrasound treatment, stable PLA dispersions with particle sizes of 200–500 nm can be obtained. In our work, after the fabrication of stable dispersions, they were washed to remove the stabilizing surfactant through centrifugation. It was found that during this process, it is possible to separate a fine fraction of PLA particles as well. 

**Figure 8:**
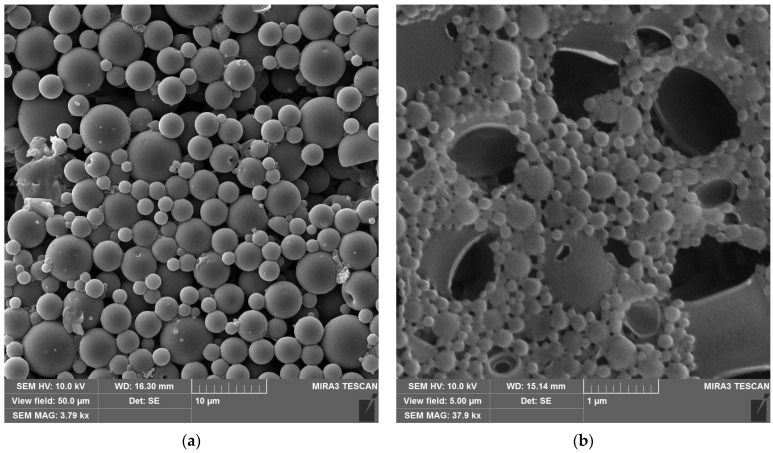
PLA suspension particles: (**a**) formed with mechanical homogenization; (**b**) formed with a combination of ultrasonic and mechanical homogenization.

**Figure 9:**
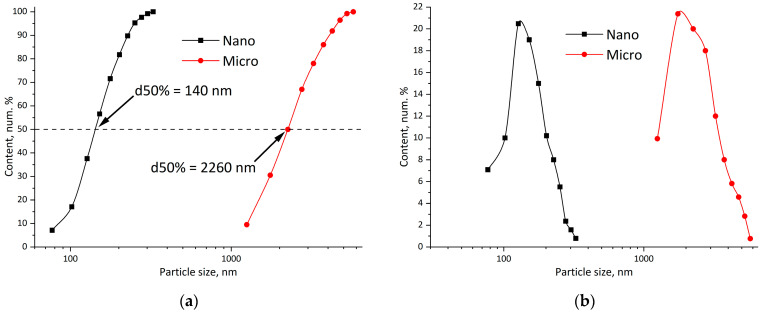
Particle size distribution of dispersions obtained by mechanical + ultrasonic dispergation (nano) and mechanical dispergation (micro): (**a**) cumulative; (**b**) differential.

Figure 8 presents comparative images of particles obtained by mechanical dispersion (Figure 8a) and by a combination of 30 min ultrasonication + centrifugal separation (Figure 8b). The samples were free of solvent and were obtained by casting the dispersion onto glass and drying at ambient temperature (20 ± 2 °C) and RH 30 ± 5%. As may be noticed, the particle size in Figure 9a,b differs by at least one order. In the first case, particles with an average size of 2.2 μm are observed, whereas in the second case, the average size is 140 nm. It may also be noted that there is a difference between micro- (Figure 8a) and nano-sized (Figure 8b) particles in the sintering of the latter even under ambient conditions, as can be seen from the images. However, complete coalescence of particles does not happen and requires further measures. 

**Figure 10:**
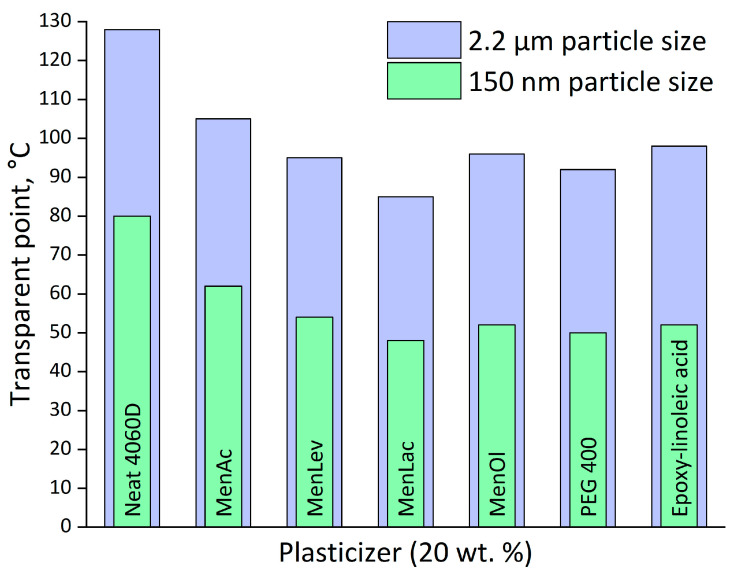
Film formation temperature (transparency point) for PLA water dispersions.

As can be seen from Figure 10, the minimum film formation temperature is significantly affected by plasticizer addition. Even the less effective MenAc decreases this value for the microparticle dispersion by 23 °C, whereas the most effective MenLac reduces it by 43 °C at a content of 20 wt. %. Particle size reduction and the following reduction in capillary diameter increase the coalescence force and, as a result, suppress the minimum transparency temperature. Particles with sizes of 2.2 µm and 140 nm differ by more than one order in size, which lowers the MFFT from 128 °C to 80 °C. The addition of plasticizers in this case is also effective: MenLac provides film formation at 48 °C, while MenOl and PEG-400 have a comparable effect. It is worth mentioning that the MFFT and T_g_ are close in this case; therefore, it may be assumed that the plasticization effectiveness is close to its theoretical threshold limited by T_g_ when the particles are sufficiently fine.

Following the trends observed in Figure 10, the combined effect of dispersion characteristics and plasticizer chemistry can be rationalized as a coupled formulation strategy governing film formation in waterborne PLA systems. A reduction in particle size leads to the formation of narrower interparticle capillaries during drying, which in turn increases the capillary pressure driving particle deformation and coalescence. This capillary contribution becomes particularly important in waterborne dispersions, where film formation is initiated by water removal and the development of capillary forces between adjacent particles. At the same time, the introduction of menthol-based plasticizers lowers the glass transition temperature of PLA, thereby enhancing polymer chain mobility at temperatures relevant to drying and film coalescence. The experimental data demonstrate that plasticized systems exhibit markedly reduced T_g_ and MFFT compared with neat PLA, indicating that the critical temperature required for interparticle fusion can be shifted toward milder processing conditions. In this framework, particle size reduction primarily amplifies capillary driving forces by decreasing the radius of interparticle liquid bridges, whereas tailored plasticization supplies the necessary thermodynamic and kinetic mobility for effective interdiffusion across particle boundaries. Although particle size and plasticizer content were not independently varied as separate parameters in this study, the observed improvements in film continuity and reduced film formation temperature support the synergistic nature of this combined approach. Overall, these results suggest that coordinated control of dispersion particle size and plasticizer chemistry offers an effective pathway for optimizing waterborne PLA coatings without resorting to aggressive processing conditions.

**Figure 11:**
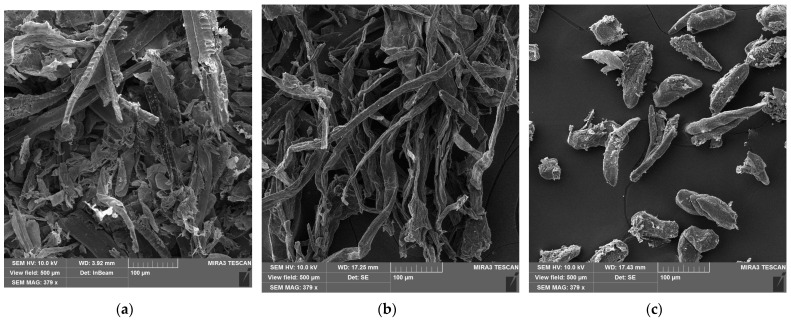
Natural fibers from agricultural waste: (**a**) sunflower seed hulls; (**b**) cornstalk; (**c**) coconut shell.

In the design of technologically mature coatings derived from disperse particles, whether aqueous dispersions or powder-based systems, the ability of particles to coalesce with other components of the formulation, particularly their capacity to wet the surface of fillers, remains a critical requirement. In the present study, fillers originating from agricultural waste, specifically sunflower seed husks, cornstalks, and coconut shells were investigated. As demonstrated in Figure 11a and Figure 11b, the first two sources enable the production of cellulose-based fibrous particles with an L/D ratio of 10.5 ± 0.5 and 11.5 ± 0.5, respectively, indicating their potential for use as reinforcing agents and shrinkage-reducing fillers. The particles produced from coconut shells (Figure 11c) (L/D ratio = 8.5 ± 0.4) exhibit characteristics more consistent with inert fillers within the system. 

**Figure 12:**
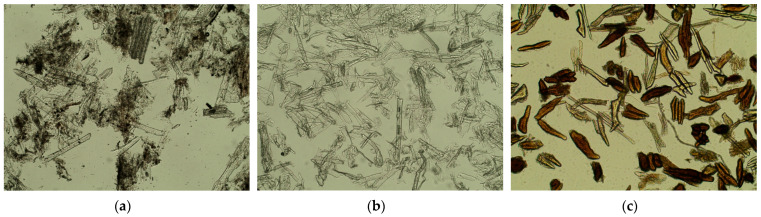
Distribution of fibers in the PLA matrix after particle sintering: (**a**) sunflower seed hulls; (**b**) cornstalk; (**c**) coconut shell.

Cellulose-based fibrous fillers possess intrinsically polar surfaces: the free surface energy coordinates at 23 °C are typically $\gamma_{S}^{D}\;$≈ 38–40 mJ/m^2^ and $\gamma_{S}^{P}$ ≈ 8.6–12.6 mJ/m^2^ (total surface energy is ≈48–51 mJ/m^2^) [39]. By contrast, polylactide (PLA) is much less polar, with PLA film coordinates $\gamma_{S}^{D}$ ≈ 32.8 mJ/m^2^ and $\gamma_{S}^{P}$ ≈ 5.8 mJ/m^2^ (total surface energy is ≈38.6 mJ/m^2^) [40]. Reducing the polarity of cellulose via silane treatments narrows this mismatch and thermodynamically improves PLA wetting/adhesion by lowering the polar contribution to the interfacial tension. Because the PLA ester backbone undergoes moisture-catalyzed hydrolysis that accelerates with temperature and humidity, causing rapid molecular-weight loss and deterioration of mechanical properties during processing and service, silanization may be a measure to limit such processes [41,42]. Moreover, reducing the polarity of cellulose surfaces suppresses interparticle hydrogen bonding and agglomeration, which lowers the melt viscosity of PLA—cellulose composites and markedly improves their processability during extrusion and molding [43]. Another remarkable effect is the reduction in interfacial water accumulation in PLA composites, thereby limiting hydrolysis-driven embrittlement and enhancing both the long-term durability and dimensional stability of the material under humid conditions [44]. In our study, the silanization of the obtained cellulose fibers helped to decrease the moisture uptake of the compositions from 3.30 to 3.02 wt. % (coconut), from 3.6 to 2.8 wt. % (cornstalk), and from 3.50 to 2.96 wt. % (sunflower seed hulls). The distribution of the fibers inside the sintered particle polylactide matrix (Figure 12) is even and without clogs (which are usual in the case of fiber–matrix incompatibility). 

In the case of cornstalk, the refined particles form a net-like interconnection, making them suitable candidates for reinforcing fillers. Although the particles derived from sunflower seed hulls exhibit a high L/D ratio, their reinforcing potential is lower than that of particles from cornstalks due to the presence of non-fibrous impurities. The coconut shell-derived particles are more suitable as fillers for PLA compositions, as they do not contain a sufficiently high fraction of fibrous elements. 

Overall, it can be stated that the obtained plasticized PLA dispersions form composite coatings during film formation in combination with cellulose-based fillers derived from agricultural waste. 

From an application-oriented perspective, the performance of the proposed waterborne PLA coatings is inherently influenced by the nature of the substrate. Non-porous and low-absorbency substrates, such as polymer films, metal foils, or coated paper, represent the most suitable cases, as they allow efficient film formation without significant loss of plasticizer into the substrate. In such systems, the reduced T_g_ and MFFT achieved through tailored plasticization enable continuous coating formation under moderate drying conditions, making them attractive for packaging or protective layers where solvent-free processing is required. In contrast, highly porous or absorbent substrates may partially uptake low-molecular-weight plasticizers, leading to local variations in composition and, consequently, altered film formation behavior. Another practical limitation arises in applications where additional thermal input is restricted or undesirable, as film formation still requires drying temperatures at or above the MFFT of the formulation. Therefore, while the present results demonstrate clear advantages for controlled, non-porous substrates under moderate heating, further formulation refinement or substrate pre-treatment would be required to extend applicability to heat-sensitive or highly porous materials.

From a sustainability perspective, the proposed approach combines several advantages at both the material and process levels. The use of waterborne PLA dispersions avoids organic solvents during coating formation, reducing volatile organic compound emissions and simplifying processing. The dichloromethane or other solvent is effectively trapped and regenerated. In addition, both PLA and the investigated menthol-based components are derived from renewable resources, aligning the formulations with current strategies for bio-based and low-carbon polymer systems. Importantly, the observed reduction in T_g_ and MFFT enables film formation at lower drying temperatures, which puts this technology at the boundary of conventional ambient-film-forming systems.

Although long-term aging or water-exposure stability was not directly assessed in this study, the results provide a framework for anticipating durability trends. Specifically, the clear differentiation between water-soluble screening plasticizers and hydrophobic plasticizers intended for final coatings allows early identification of formulations with higher potential resistance to plasticizer loss and property drift over time. Therefore, the present work establishes a sustainable formulation concept and a rational screening strategy, while aging and long-term performance evaluation represent the next stage of ongoing research.

## 4. Conclusions

This study demonstrates a viable approach to sustainable waterborne PLA coatings by combining particle size reduction with tailored plasticization. For the first time, water-insoluble DES-type plasticizers for PLA were synthesized, including menthol–oleic acid (1:1), which overcomes the leaching limitations associated with water-soluble deep eutectic solvents.

Particle size reduction from approximately 2.2 µm (mechanical dispersion) to 140 nm (mechanical plus ultrasonication) decreased the minimum film formation temperature (MFFT) from 128 °C to 80 °C. Plasticization further reduced the MFFT to 48 °C using menthol–lactic acid at 20 wt. %. Differential scanning calorimetry confirmed corresponding glass transition temperature depressions of −17 °C (PEG-400), −10 °C (epoxidized linoleic acid), and −6 °C (menthol–oleic acid), consistent with compatibility trends predicted by Hansen solubility parameters.

Moreover, the dispersions enabled the fabrication of crack-free composite coatings incorporating cellulose-based fillers derived from agricultural residues. Fiber dimensions (L/D = 10.5–11.5) and silanization treatment improved compatibility and reduced moisture uptake (e.g., cornstalks from 3.6 wt. % to 2.8 wt. %). These results highlight the potential of plasticized PLA dispersions for energy-efficient, low-temperature curing and the integration of bio-derived reinforcement into waterborne coatings.

As a representative formulation example for application, the nano-sized PLA dispersion combined with 20 wt. % menthol–lactic acid achieved low-temperature film formation (48 °C). This binder can be used to produce crack-free composite coatings with cellulose fibers from agricultural residues. Together, this case demonstrates a practical route to low-temperature curing waterborne PLA coatings that integrate bio-derived reinforcement while maintaining performance-relevant integrity.

Overall, this work establishes a scalable foundation for low-VOC, renewable PLA-based coating materials, supporting future developments in sustainable polymer technologies.

## Figures and Tables

**Table 1:** Hansen solubility parameters of plasticizers and PLA polymer.

Title 1	δd	δp	δh	R_0_	R_a_	RED	Ref.
Menthol–levulinic acid 1:1 (MenLev)	17.15	5.17	10.96		6.7	0.79	[31]
Menthol–acetic acid 1:1 (MenAc)	16.88	4.15	11.60		7.8	0.92	[31]
Menthol–lactic acid 1:1 (MenLac)	16.7	2.5	8.8		7.8	0.92	-
Menthol–oleic acid 1:1 (MenOl)	16.8	5.0	9.4		5.8	0.68	-
Epoxy oleic acid	16.6	11.1	9.8		3.6	0.42	[28]
Epoxy linoleic acid	16.6	11.4	10.5		4.4	0.51	[28]
PEG-400	14.6	7.5	9.4		5.4	0.64	[32]
PLA 4060D	16.5	9.9	6.4	8.5			[28]

## Data Availability

The original contributions presented in this study are included in the article. Further inquiries can be directed to the corresponding author.

## Abbreviations

The following abbreviations are used in this manuscript:

PLA	Polylactide
MFFT	Minimum film formation temperature
HDES	Hydrophobic deep eutectic solvent
NMR	Nuclear magnetic resonance
FTIR	Fourier-transform infrared spectroscopy
RED	Relative energy difference
PEG	Polyethylene glycol
SDS	Sodium dodecyl sulfate
DSC	Differential scanning calorimetry
